# DFNA20/26 and Other ACTG1-Associated Phenotypes: A Case Report and Review of the Literature

**DOI:** 10.3390/audiolres11040052

**Published:** 2021-10-18

**Authors:** Ugo Sorrentino, Chiara Piccolo, Chiara Rigon, Valeria Brasson, Eva Trevisson, Francesca Boaretto, Alessandro Martini, Matteo Cassina

**Affiliations:** 1 Clinical Genetics Unit, Department of Women’s and Children’s Health, University of Padova, 35128 Padova, Italy; chiara.piccolo@aopd.veneto.it (C.P.); chiara.rigon@unipd.it (C.R.); valeria.brasson@aopd.veneto.it (V.B.); eva.trevisson@unipd.it (E.T.); francesca.boaretto@aopd.veneto.it (F.B.); 2 Padova University Research Center “I–APPROVE, International Auditory Processing Project in Venice”, “Santi Giovanni e Paolo” Hospital, 30122 Venice, Italy; alessandromartini@unipd.it

**Keywords:** *ACTG1*, DFNA20, DFNA26, gamma-actin, non-syndromic hearing loss, NGS

## Abstract

Since the early 2000s, an ever-increasing subset of missense pathogenic variants in the *ACTG1* gene has been associated with an autosomal-dominant, progressive, typically post-lingual non-syndromic hearing loss (NSHL) condition designed as DFNA20/26. *ACTG1* gene encodes gamma actin, the predominant actin protein in the cytoskeleton of auditory hair cells; its normal expression and function are essential for the stereocilia maintenance. Different gain-of-function pathogenic variants of *ACTG1* have been associated with two major phenotypes: DFNA20/26 and Baraitser–Winter syndrome, a multiple congenital anomaly disorder. Here, we report a novel *ACTG1* variant [c.625G>A (p. Val209Met)] in an adult patient with moderate-severe NSHL characterized by a downsloping audiogram. The patient, who had a clinical history of slowly progressive NSHL and tinnitus, was referred to our laboratory for the analysis of a large panel of NSHL-associated genes by next generation sequencing. An extensive review of previously reported *ACTG1* variants and their associated phenotypes was also performed.

## 1. Introduction

*ACTG1* gene (MIM *102560) encodes gamma actin, a highly conserved cytoskeletal protein that plays essential roles in many processes of eukaryotic cell biology [1]. Gamma actin is the predominant actin protein in the cytoskeleton of auditory hair cells, where it is found in stereocilia, the cuticular plate, and adherens junctions [2]; its normal expression and function are essential for the stereocilia maintenance but are not required for auditory hair cell development [3,4].

Different gain-of-function pathogenic variants of *ACTG1* have been associated with two phenotypes: autosomal dominant non-syndromic sensorineural deafness 20/26 (DFNA20/26, MIM #604717) and Baraitser–Winter syndrome (MIM #614583) [5,6]. DFNA20/26 is characterized by progressive post-lingual hearing loss (HL) with an onset ranging usually from the first to the third decade of life; high frequencies are the first to be affected, and audiograms typically show a sloping configuration. The rate of progression is variable but usually results in profound deafness by the 4th to 6th decade. Baraitser–Winter syndrome is characterized by typical craniofacial dysmorphisms and intellectual disability that may be associated with congenital anomalies of the brain, iris or retinal coloboma, HL, muscle wasting, heart defects, and renal malformations; the expressivity of the disorder is variable [7]. Unlike non-syndromic sensorineural deafness, Baraitser–Winter syndrome (MIM #243310) can also be caused by mutations in *ACTB*, the gene encoding beta actin [6].

In this paper, we describe an additional *ACTG1* variant in a patient with an auditory phenotype of DFNA20/26 and perform a review of the literature.

## 2. Materials and Methods

A retrospective analysis of genetic and audiological data of a patient with DFNA20/26 evaluated at the Clinical Genetics Units of the University Hospital of Padova (Italy) was performed. The case was selected from among patients who were referred for the molecular analysis by next generation sequencing (NGS) of a panel of 79 genes associated with HL (Supplementary Table S1). The multi-gene NGS analysis was performed on genomic DNA extracted from peripheral leukocytes. The coding regions of the selected genes were isolated and captured using the SureSelect Target Enrichment systems (Agilent Technologies, Santa Clara, CA, USA); indexed DNA fragments libraries were generated according to the manufacturer’s protocol and sequenced on a NextSeq 550 instrument (Illumina, San Diego, CA, USA). Variant calling and annotation, as well as a subsequent bioinformatic analysis to detect Copy Number Variations, were performed using the SureCall software (Agilent Technologies). DFNB1-associated variations were excluded by NGS (coding region of *GJB2* exon 2), Sanger sequencing (*GJB2* exon 1 and exon-intron boundaries), and multiplex PCR (method described by Castillo et al. [8] to detect two large deletions encompassing part of the *GJB6* gene, del(GJB6-D13S1830) and del(GJB6-D13S1854)). The variant in *ACTG1* gene was reported according to the RefSeq transcript NM_001614.5; the predicted change on the encoded protein was reported according to the RefSeq protein NP_001605.1. The audiological assessments and the genetic tests were part of the routine diagnostic and follow-up procedures. Written informed consent for molecular genetic studies was collected.

A retrospective review of the literature on *ACTG1* variants and DFNA20/26 phenotype was performed. Pertinent English language articles were searched in PubMed (https://pubmed.ncbi.nlm.nih.gov/) and Web of Science (https://www.webofscience.com/) using the key words ‘*ACTG1*’, ‘DFNA’, ‘DFNA20/26’, ‘DFNA20’, and ‘DFNA26’; the last search was performed on 31 July 2021. Demographic and genetic data, characteristics of HL, presence of tinnitus, and vestibular anomalies were extracted from previously reported patients. Additional pathogenic or likely pathogenic variants associated with DFNA20/26 were searched in mutation databases, including “Deafness Variation Database” (https://deafnessvariationdatabase.org/), LOVD—Leiden Open Variation Database (https://www.lovd.nl/), ClinVar (https://www.ncbi.nlm.nih.gov/clinvar/), and HGMD—Human Gene Mutation Database (http://www.hgmd.cf.ac.uk/). *ACTG1* variants identified in patients without phenotype details were excluded from the review analysis.

## 3. Results

### 3.1. Case Description

**Figure 1:**
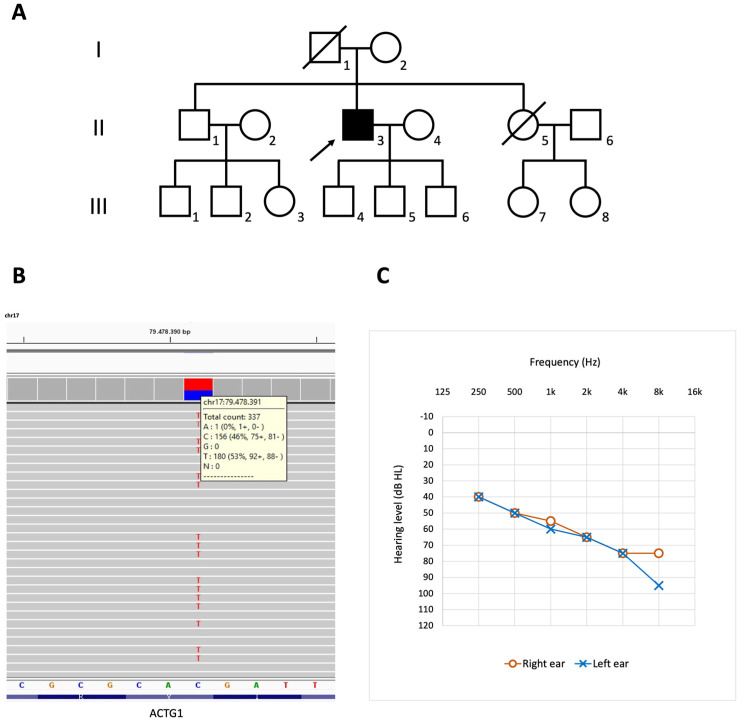
(**A**) family tree of our patient (II,3). (**B**) NGS analysis showing the cytosine (**C**) to thymine (T) substitution at nucleotide 79,478,391 of chromosome 17 [hg19]; this variation corresponds to the c.625G>A variant in exon 4 of *ACTG1*; it was detected in approximately 50% of the total number of *reads* (337) at position 79,478,391, indicating a heterozygous genotype. (**C**) Audiogram of the proband carrying the c.625G>A p.(Val209Met) variant in *ACTG1* (at age 57 years).

A 57-year-old male patient with HL was referred to our institution for genetic testing. He was the second child of healthy non-consanguineous Italian parents, and his family history was negative for neurologic disorders, deafness, developmental delay, and congenital malformations (Figure 1A). The proband’s phenotype was characterized by HL associated with tinnitus, with an onset age of approximately 6 years. Language development was completely normal, and no additional health problems were reported except for myopia and astigmatism. The patient reported slow progression of HL and started to wear hearing aids in his late 30s. At the time of our evaluation, he showed a moderate-severe HL, with a characteristic downsloping audiogram (Figure 1C). The multi-gene panel testing revealed the following heterozygous variant in exon 4 of *ACTG1* gene: c.625G>A [p.(Val209Met)] (Figure 1B). Although several features might support its possible pathogenicity, this nucleotide change still has to be considered as a variant of uncertain significance according to the hearing loss-specific rules of the ACMG Classification [9,10]. Unfortunately, no other family members accepted to be tested to evaluate the segregation of the detected variant.

### 3.2. Literature Review

A total of 36 missense *ACTG1* variants were previously reported in 48 DFNA20/26 families with a sufficient delineation of patients’ phenotype (Table 1). Three variants are located in exon 2; 8 in exon 3; 12 in exon 4; 9 in exon 5; and 4 in exon 6. Most of the variants were observed in only one family, and only eight variants were recurrent: c.353A>T (p.Lys118Met) in four families from USA, Japan, and China; c.94C>T (p.Pro32Ser) in three families from Korea and China; c.266C>T (p.Thr89Ile) in three families from USA and Japan; c.548G>A (p.Arg183Gln) in two families from Spain and Italy; c.721G>A (p.Glu241Lys) in two families from Spain and Japan; c.791C>T (p.Pro264Leu) in two families from USA and Japan; c.914T>C (p.Met305Thr) in two families from Korea and Japan; and c.994C>T (p.Pro332Ser) in two families from Japan.

In most families, HL showed the typical characteristics of DFNA20/26: a progressive, post-lingual HL with variable age at onset, ranging from the first to the third decades of life; high frequencies were the first to be affected and audiograms typically showed a sloping configuration; the rate of progression was variable but often resulted in severely profound deafness by the 6th decade. Few cases with a pre-lingual onset (including congenital HL) or with a slower progression of HL were reported, underlining the extreme clinical variability of the phenotype, even in patients carrying the same *ACTG1* variants. However, other additional genetic and/or non-genetic factors may have contributed to the pathogenesis of HL in those patients with a congenital or early onset of the disorder.

Tinnitus was inconsistently reported in patients with 12 different *ACTG1* variants, suggesting that its pathogenesis may not be related to the genotype. The same applies for vertigo and other vestibular symptoms, which were inconsistently reported in patients with seven different *ACTG1* variants; however, such symptomatology may have been underreported, since most patients did not undergo vestibular function tests.

Additional details are shown in Table 1.

## 4. Discussion

HL is the most prevalent sensory impairment in the human species, with an estimated incidence of 1–3 in 1000 live births [36]. A classic Mendelian genetic etiology was recognized in approximately 50–60% of cases of HL, which for the most part occur as non-syndromic forms (NSHL). NSHL is characterized by remarkable genetic heterogeneity, as pathogenic variants have so far been reported in more than 100 genes.

In the year 2000, by means of linkage analysis in a three-generation Midwestern United States family, Morell et al. managed to map, for the first time, a novel HL-related locus in the 17q25 chromosomal region, designated as DFNA20; the phenotype was characterized by bilateral, sloping, progressive, sensorineural HL, that in some of the cases was detectable since the early teens but became generally evident in the third decade [37,38]. In the same year, Yang and Smith reported two unrelated families that presented with a progressive autosomal dominant HL condition (designed as DFNA26) that mapped to chromosome 17q25 as well [39]. DeWan et al. described yet another American family in which affected members had sloping audiograms with mid- and high-frequency HL, which progressed to HL that affected all frequencies; the phenotype was more accurately pinpointed at the 17q25.3 locus, concluding that DFNA20 and DFNA26 were probably the same disorder [40]. The identification of the deafness causative gene in these four families was finally performed by Zhu et al. who demonstrated that missense pathogenic mutations in the gamma actin gene (*ACTG1*) were associated with a distinguished autosomal dominant, progressive, sensorineural HL phenotype [5]. In the following 6 years, additional *ACTG1* variants were described in DFNA20/26 families in The Netherlands, Norway, China, and Spain [15,19,20,28,33].

In recent years, the worldwide capability of investigating genetically heterogeneous diseases such as HL has significantly improved thanks to the implementation of next-generation sequencing (NGS) technologies, which allow the parallel sequencing of large panels of disease-relevant genes and thus the identification of a considerable number of genomic variants, both in previously known disease-associated genes and in new candidates.

To date, a total of 36 mutations in *ACTG1* have been reported from 48 unrelated families with non-syndromic hearing loss (NSHL), allowing to better delineate the DFNA20/26 phenotype and its variability. The *ACTG1* variant we have detected [c.625G>A p.(Val209Met)] has never been reported in the literature nor in the available databases. In addition, the variant is very rare in the general population (it was not found in databases aggregating exome and genome sequencing data from large-scale sequencing projects), and multiple lines of computational evidence support its deleterious effect. Although these features might support its possible pathogenicity, this novel nucleotide change still has to be considered as a variant of uncertain significance according to the hearing loss-specific rules of the ACMG Classification [9,10]. The analysis of the variant segregation in other family members, associated with an in-depth analysis of their audiological phenotype, could have added important information and allowed a better classification of the variant; however, to date no other proband’s relatives agreed to be tested.

According to the review of previously reported cases, *ACTG1*-related NSHL is usually post-lingual and progressive. The age at onset is variable, with most cases presenting in the first or second decade of life; cases with late-onset HL (up to the 5th decade) are reported, but at least in some of these we cannot exclude an earlier onset of the auditory deficit, associated with slow progression rate and a clinically undiagnosed mild HL. HL typically affects the higher frequencies at first and then progresses to all frequencies, with audiograms showing a characteristic sloping configuration; the rate of HL deterioration varies among families and patients (from 1 dB/year to 6 dB/year) [13,14,15], but it is more rapid for the higher frequencies than the lower ones [13]. HL is usually severe to profound by age 60 years, with the sloping configuration of the audiogram usually maintained.

In our patient, the characteristics of HL (age at onset, audiogram configuration, and progression) are comparable with those previously reported in DFNA20/26 patients; however, only a moderate-severe HL was observed in our 57-year-old patient, indicating a slower progression of HL deterioration than that described in the majority of patients.

In addition, tinnitus was one the major symptoms experienced by our proband since the onset of HL; tinnitus has been reported in several patients with DFNA20/26 in the literature [13], but at the moment there are insufficient data to draw any conclusions regarding its prevalence among patients with DFNA20/26 (details regarding this symptom are lacking for a relevant number of patients reported in the literature) and the possible pathogenic mechanisms related to *ACTG1* mutations. However, the available data do not support a correlation with the severity of HL nor with the location of the variant in specific subdomains of the protein encoded by *ACTG1*.

Vertigo and vestibular symptoms have been inconsistently reported in patients with DFNA20/26. Even if our patient did not experience such symptomatology, it is not surprising that vestibular symptoms may be part of the DFNA20/26 phenotype, given that gamma actin is also present in the stereocilia of vestibular hair cells [14,41].

When HL is genetically determined, the auditory outcome after cochlear implantation (CI) can be generally predicted according to the molecular mechanisms associated with the known causative gene. Specifically, a better auditory performance is expected in the case of an intra-cochlear etiology. Since gamma actin participates in the maintenance of the hair cells of the cochlea, patients with DFNA20/26 are thus generally expected to exhibit a good outcome from CI. The data available in this regard are limited, yet encouraging, as some authors observed good CI performance in affected individuals [14,27]. In addition to CI, electric-acoustic stimulation (EAS) has also been speculated to be effective in the therapy of *ACTG1*-associated HL. Even though very few cases have so far been reported in the literature, it should be noted that all of them exhibited a favorable outcome [14,29,42].

Gamma actin is the predominant actin protein in the cytoskeleton of auditory hair cells, where it is found in stereocilia, the cuticular plate, and adherens junctions [2]; its normal expression and function are essential for the stereocilia maintenance but are not required for auditory hair cell development [3,4]. The molecular pathogenic effect of missense variants detected in the first families described in the literature was evaluated using a variety of biochemical analyses, studies with yeast and hair cells of mouse cochlear explants, and Actg1 knock-in mouse models [4,19,43,44,45,46]. These results suggest a gain-of-function mode of pathogenesis, with missense mutations giving rise to a series of small changes that result in misregulation of actin assembly and dynamics; these alterations progressively damage auditory hair cells but are tolerated in other cell types [4]. Among inner ear hair cells, cochlear cells seem to be more susceptible to damage than the vestibular ones, given that vestibular signs and symptoms are reported in a minority of cases with DFNA20/26.

*ACTG1* variants have also been associated to Baraitser–Winter cerebrofrontofacial (BWCFF) syndrome, a multiple congenital anomaly disorder characterized by typical craniofacial features and intellectual disability; many, though not all, of the affected patients may have iris or retinal coloboma, sensorineural HL of variable degree, muscle wasting, seizures, congenital heart defects, and renal malformations [6,7,47]. HL can be progressive and has been estimated to be present in approximately 40–50% of cases with BWCFF syndrome caused by an *ACTG1* mutation [7,47]. Unlike DFNA20/26, BWCFF syndrome is typically caused by de novo mutations; in fact, the complex phenotype of the syndrome, often including intellectual disability, is associated with a severely reduced genetic fitness. Just as *ACTG1* variants leading to DFNA20/26, those leading to BWCFF syndrome most probably determine a gain-of-function effect [6,7], but the exact pathogenic mechanism is yet to be clarified. Anyway, the spectrum of pathogenic variants observed in patients with DFNA20/26 does not overlap with the one observed in patients with a typical BWCFF syndrome (Supplementary Tables S2 and S3) [6,7,47,48,49,50,51,52,53,54,55,56,57,58,59,60,61,62,63,64,65,66]. In addition, the results of preliminary studies with lymphoblastoid cell lines from BWCFF syndrome patients, as well as the results of in vitro and in vivo studies on DFNA20/26-associated variants, suggest that the phenotypic impact of missense mutations may differ in a mutation-specific way; the available functional studies highlighted that each *ACTG1* variant led to specific alterations in terms of actin organization and polymerization, interaction with actin-binding proteins, and cell morphology [4,6,7,19,43,44,45,46]. Given that HL is a common feature of BWCFF syndrome, DFNA20/26 may represent the mild end and BWCFF syndrome the severe end of a wide spectrum of *ACTG1*-associated phenotypes [6]. This hypothesis is supported by the description of few families segregating mutations in *ACTG1*, in which borderline phenotypes were observed. Miyagawa et al. described a family (Family 4) in which the 7-year-old proband was affected by non-congenital, progressive HL associated with a developmental disorder, including a delay in language development; his 3-year-old sister had mild HL involving higher frequencies, but no developmental anomalies were reported; their mother displayed a post-lingual, progressive HL and a movement disorder at ages 10 and 24, attributed to a Moyamoya disease [14]. Kemerley et al. identified a three-generation pedigree in which family members with mild BWCFF syndrome (HL, learning disability, bilateral coloboma of the retina and iris in patient 2; HL and seizure disorder in patient 3) co-existed with a member with likely isolated HL (patient 1) [52]. Moreover, Lee et al. reported on a family in which the proband presented with congenital, bilateral severe HL, associated with complete bilateral cleft lip and palate and normal brain imaging; his father exhibited profound pre-lingual deafness without any other health problems (brain imaging was normal) nor dysmorphic craniofacial features [11]. Even if we cannot exclude that non-HL features in these patients may be coincidental, it is tempting to conclude that their phenotype may be classified as a mild form of BWCFF syndrome. In fact, it is important to note that BWCFF phenotype due to *ACTG1* variants is usually milder than the phenotype due to *ACTB* variants; particularly, BWCFF patients with *ACTG1* mutations present with mild or unspecific facial features and therefore could be unrecognized [47,48]. Moreover, even though all the variants listed in Table 1 were detected in patients reported to have NSHL, additional phenotypic features cannot be excluded in young children with sporadic HL and a short follow-up. Finally, it is interesting to note that a recurrent *ACTG1* variant [c.209C>T p.(Pro70Leu)] has been reported also in patients affected by isolated ocular coloboma, expanding the *ACTG1*-associated phenotypic spectrum [55].

## 5. Conclusions

NGS technologies greatly expanded the capability to identify candidate pathogenic genomic variants in affected individuals, but the interpretation of the single variants still remains a challenging task. In the specific instance of *ACTG1*-associated HL, a further level of complexity arises, as highlighted by this review, due to the heterogeneity of the reported clinical manifestations and the current uncertainty about the precise molecular and cellular mechanisms that determine non-syndromic and syndromic phenotypes. As in other similar instances, functional studies proved to be the better suited tool to determine the actual pathogenicity of *ACTG1* genomic variants and may even allow to comprehend, in the long term, the exact pathogenic mechanisms behind non-syndromic and syndromic *ACTG1*-associated HL phenotypes.

## Figures and Tables

**Table 1:** *ACTG1* variants reported in DFNA20/26 families (including the novel variant described in the present study) and associated clinical characteristics.

Exon ^1^	Nucleotide Change ^1^	Protein Change ^1^	Protein Subdomain ^2^	Number of Families	HL Onset	Age at HL Onset ^3^	High Frequencies More Severily Affected	HL Progression	Tinnitus ^4^	Vertigo and Other Vestibular Symptoms ^4^	Other Associated Features	References
2	c.94C>T	p.Pro32Ser	1	3	Pre-lingual (congenital)/Post-lingual	Birth–3 years	+	NA	−	−	Complete bilateral CLP in a patient ^5^	Lee et al. (2018) [11], Wang et al. (2018) [12]
2	c.102C>G	p.Ile34Met	2	1	Post-lingual	15–18 years	+	+	+	+	−	Miyajima et al. (2020) [13]
2	c.110G>A	p.Arg37His	2	1	Post-lingual	10 years	+	+	+	+	−	Miyajima et al. (2020) [13]
3	c.142G>C	p.Gly48Arg	2	1	Post-lingual	6–11 years	+	+	+	−	−	Miyagawa et al. (2015) [14], Miyajima et al. (2020) [13]
3	c.151G>A	p.Asp51Asn	2	1	Post-lingual (?)	1st decade	+	+	+	+	−	de Heer et al. (2009) [15]
3	c.197C>T	p.Thr66Ile	2	1	NA	NA (Early-onset)	+	NA	NA	NA	−	Morgan et al. (2018) [16]
3	c.244A>T	p.Met82Leu	1	1	Post-lingual	Adulthood	NA	NA	NA	NA	−	Sloan-Heggen et al. (2016) [17]
3	c.246G>A	p.Met82Ile	1	1	NA	NA	+	NA	−	−	−	Miyajima et al. (2020) [13]
3	c.266C>T	p.Thr89Ile	1	3	Post-lingual	Early teens/Adulthood	+	+	+	−	−	Zhu et al. (2003) [5], Sloan-Heggen et al. (2016) [17], Miyajima et al. (2020) [13]
3	c.353A>T	p.Lys118Met	1	4	Post-lingual	15–26 years	+	+	+	+	−	Zhu et al. (2003) [5], Miyagawa et al. (2015) [14], Wang et al. (2018) [18], Miyajima et al. (2020) [13]
3	c.354G>C	p.Lys118Asn	1	1	Post-lingual	3rd decade	+	+	NA	NA	−	Morín et al. (2009) [19]
4	c.364A>G	p.Ile122Val	1	1	Post-lingual	6 years	+	+	NA	−	−	Liu et al. (2008) [20]
4	c.434C>G	Ser145Cys	3	1	Pre-lingual (congenital)	Birth	NA	NA	NA	NA	−	Cabanillas et al. (2018) [21]
4	c.434C>T	p.Ser145Phe	3	1	Pre-lingual (congenital)	Birth	NA	+	NA	NA	−	Baux et al. (2017) [22]
4	c.485C>T	p.Thr162Met	3	1	Post-lingual	NA (Late onset)	NA	NA	NA	NA	−	Miyagawa et al. (2013) [23]
4	c.493A>G	p.Ile165Val	3	1	Post-lingual	11 years	+	+	−	−	−	Miyajima et al. (2020) [13]
4	c.542C>G	p.Ala181Gly	4	1	Post-lingual	Childhood	NA	NA	NA	NA	−	Sloan-Heggen et al. (2016) [17]
4	c.548G>A	p.Arg183Gln	4	2	Post-lingual (?)	Childhood (early-onset)	+	NA	NA	NA	−	Cabanillas et al. (2018) [21], Morgan et al. (2018) [16]
4	c.559G>C	p.Asp187His	4	1	Pre-lingual	1st year	NA	NA	NA	NA	−	Baek et al. (2012) [24]
4	c.625G>A	p.Val209Met	4	1	Post-lingual	6 years	+	+	+	−	−	This study
4	c.638A>G	p.Lys213Arg	4	1	Post-lingual	2nd decade	+	+	−	NA	−	Yuan et al. (2016) [25]
4	c.721G>A	p.Glu241Lys	4	2	Post-lingual	3–14 years	+	+	−	−	Developmental disorder; Moyamoya disease ^6^	Morín et al. (2009) [19], Miyagawa et al. (2015) [14], Miyajima et al. (2020) [13]
4	c.791C>T	p.Pro264Leu	4	2	Post-lingual	2nd decade	+	+	+	+	−	Zhu et al. (2003) [5], Miyajima et al. (2020) [13]
4	c.802G>A	p.Gly268Ser	4	1	Post-lingual	10–45 years	+	+	NA	NA	−	Mutai et al. (2013) [26]
5	c.823C>T	p.His275Tyr	3	1	Post-lingual	34 years	+	+	+	−	−	Miyajima et al. (2020) [13]
5	c.830C>T	p.Thr277Ile	3	1	Post-lingual	1st–2nd decades	+	+	NA	NA	−	Liu et al. (2019) [27]
5	c.833C>T	p.Thr278Ile	3	1	Post-lingual	5–25 years	+	+	NA	+	−	van Wijk et al. (2003) [28]
5	c.847A>G	p.Met283Val	3	1	Post-lingual	Adulthood	NA	NA	NA	NA	−	Morgan et al. (2018) [16]
5	c.848T>C	p.Met283Thr	3	1	Post-lingual (?)	Childhood (early-onset)	NA	NA	NA	NA	−	Cabanillas et al. (2018) [21]
5	c.895C>G	p.Leu299Val	3	1	Post-lingual	1st–5th decades	+	+	+	−	−	Miyagawa et al. (2013) [29], Miyagawa et al. (2015) [14], Miyajima et al. (2020) [13]
5	c.914T>C	p.Met305Thr	3	2	Post-lingual	6 years	+	+	+	−	−	Park et al. (2013) [30], Miyajima et al. (2020 [13]
5	c.946G>A	p.Glu316Lys	3	1	Post-lingual	NA	+	+	NA	−	−	Wei et al. (2014) [31]
5	c.974T>A	p.Met325Lys	3	1	Pre-lingual (congenital) ^7^	Birth	+	NA	NA	NA	−	Vona et al. (2014) [32]
6	c.994C>G	p.Pro332Ala	3	1	Post-lingual	Before early teens/2nd decade	+	+	NA	−	−	Zhu et al. (2003) [5]
6	c.994C>T	p.Pro332Ser	3	2	Post-lingual	35–59 years	+	+	+	−	−	Miyajima et al. (2020) [13]
6	c.1045C>A	p.Leu349Met	1	1	Pre-lingual (congenital)	Birth	NA	NA	NA	NA	NA	Sloan-Heggen et al. (2016) [17]
6	c.1109T>C	p.Val370Ala	1	1	Post-lingual	1st–2nd decades	+	+	NA	+	−	Rendtorff et al. (2006) [33]

^1^ Exon numbering and nucleotide changes of *ACTG1* gene was reported according to the RefSeq transcript NM_001614; the predicted protein change based on DNA data was reported according to the RefSeq protein NP_001605.1. ^2^ Subdomain 1 (residues 1–32, 70–144, and 338–372), subdomain 2 (residues 33–69), subdomain 3 (residues 145–180, and 270–337), subdomain 4 (residues 181–269) [34,35]. ^3^ The range of age at HL onset is reported, considering all the individuals with the specific *ACTG1* variant for whom the information is available. ^4^ Tinnitus, vertigo, and other vestibular symptoms were considered present (+) if at least one patient with the specific *ACTG1* variant was reported to manifest the symptom. ^5^ Complete bilateral CLP in a patient (Lee et al., 2018) [11]; no associated anomalies in two young children described by Wang et al. (2018) [12]. ^6^ Developmental disorder in a 7-year-old boy (absent in the younger sister with HL); Moyamoya disease (in their mother) (Miyagawa et al., 2015; Miyajima et al., 2020) [13,14]. No associated anomalies in the family described by Morín et al. (2009) [19]. ^7^ Father is completely deaf; mother has profound HL. Parents were not tested for the *ACTG1* variant. NA: Information Not Available; CLP: Cleft lip and palate.

## Data Availability

Not applicable.

## Supplementary Materials

The following are available online at https://www.mdpi.com/article/10.3390/audiolres11040052/s1, Table S1: Hearing loss genes analyzed by Next Generation Sequencing. Table S2. Pathogenic and likely pathogenic *ACTG1* variants reported in patients with Baraitser-Winter syndrome and/or associated congenital anomalies. Table S3. *ACTG1* variants of uncertain significance reported in patients with Baraitser-Winter syndrome and/or associated congenital anomalies (only variants with MAF = 0 in gnomAD v2.1.1 are reported).
