# Supplementary material for: DFNA20/26 and Other ACTG1-Associated Phenotypes: A Case Report and Review of the Literature
Source: Audiol Res. 2021 Oct 18;11(4):582–93. doi: 10.3390/audiolres11040052 (PMC8544197; doi:10.3390/audiolres11040052)
Supplement: Supplementary file 1 [file audiolres-11-00052-s001.zip › audiolres-1399936-supplementary.pdf]

Table S1: Hearing loss genes analyzed by Next Generation Sequencing.

Gene	Gene MIM number	Phenotype
ACTG1	102560	Deafness autosomal dominant 20/26
ADGRV1	602851	Usher syndrome type 2C
CCDC50	611051	Deafness autosomal dominant 44
CDH23	605516	Deafness autosomal recessive 12 / Usher syndrome type 1D / Usher syndrome type 1D/F digenic
CEACAM16	614591	Deafness autosomal recessive 113 / Deafness autosomal dominant 4B
CIB2	605564	Deafness autosomal recessive 48 / Usher syndrome type IJ
CISD2	611507	Wolfram syndrome 2
CLDN14	605608	Deafness autosomal recessive 29
CLRN1	606397	Usher syndrome type 3A
COCH	603196	Deafness autosomal recessive 110 / Deafness autosomal dominant 9
COL11A1	120280	Deafness autosomal dominant 37 / Stickler syndrome type II
COL11A2	120290	Deafness autosomal recessive 53 / Deafness autosomal dominant 13 / Otospondylomegaepiphyseal dysplasia
COL2A1	120140	Stickler syndrome type I
COL4A3	120070	Alport syndrome autosomal recessive / Alport syndrome autosomal dominant
COL4A4	120131	Alport syndrome autosomal recessive
COL4A5	303630	Alport syndrome X-linked
COL9A1	120210	Stickler syndrome type IV
COL9A2	120260	Stickler syndrome type V
CRYM	123740	Deafness autosomal dominant 40
DIABLO	605219	Deafness autosomal dominant 64
DIAPH1	602121	Deafness autosomal dominant 1
DIAPH3	614567	Auditory neuropathy autosomal dominant 1
ESPN	606351	Deafness autosomal recessive 36 / Deafness neurosensory without vestibular involvement autosomal dominant / Usher syndrome type 1M
EYA4	603550	Deafness autosomal dominant 10
FOXI1	601093	Enlarged vestibular aqueduct
GATA3	131320	Hypoparathyroidism, sensorineural deafness, and renal dysplasia
GIPC3	608792	Deafness autosomal recessive 15
GJB2	121011	Deafness autosomal recessive 1A / Deafness digenic GJB2/GJB6 / Deafness autosomal dominant 3A
GJB3	603324	Deafness digenic GJB2/GJB3 / Deafness autosomal dominant 2B
GJB6	604418	Deafness autosomal recessive 1B / Deafness digenic GJB2/GJB6 / Deafness autosomal dominant 3B
GRHL2	608576	Deafness autosomal dominant 28
GRXCR1	613283	Deafness autosomal recessive 25
GSDME	608798	Deafness autosomal dominant 5
HARS1	142810	Usher syndrome type 3B
HGF	142409	Deafness autosomal recessive 39
KCNJ10	602208	Enlarged vestibular aqueduct digenic
KCNQ4	603537	Deafness autosomal dominant 2A
LOXHD1	613072	Deafness autosomal recessive 77
MIR96	611606	Deafness autosomal dominant 50

Gene	Gene MIM number	Phenotype
MITF	156845	Waardenburg syndrome type 2A
MSRB3	613719	Deafness autosomal recessive 74
MYH14	608568	Deafness autosomal dominant 4A
MYH9	160775	Deafness autosomal dominant 17
MYO15A	602666	Deafness autosomal recessive 3
MYO6	600970	Deafness autosomal recessive 37 / Deafness autosomal dominant 22
MYO7A	276903	Deafness autosomal recessive 2 / Deafness autosomal dominant 11 / Usher syndrome type 1B
OSBPL2	606731	Deafness autosomal dominant 67
OTOA	607038	Deafness autosomal recessive 22
OTOF	603681	Deafness autosomal recessive 9 / Auditory neuropathy autosomal recessive 1
OTOG	604487	Deafness autosomal recessive 18B
OTOGL	614925	Deafness autosomal recessive 84B
P2RX2	600844	Deafness autosomal dominant 41
PAX3	606597	Waardenburg syndrome type 1 and type 3
PCDH15	605514	Deafness autosomal recessive 23 / Usher syndrome type 1F / Usher syndrome type 1D/F digenic
PDZD7	612971	Deafness autosomal recessive 57 / Usher syndrome type IIC
PJVK	610219	Deafness autosomal recessive 59
POU3F4	300039	Deafness X-linked 2
POU4F3	602460	Deafness autosomal dominant 15
PRPS1	311850	Deafness X-linked 1
PTPRQ	603317	Deafness autosomal recessive 84A / Deafness autosomal dominant 73
RDX	179410	Deafness autosomal recessive 24
SIX1	601205	Deafness autosomal dominant 23
SLC17A8	607557	Deafness autosomal dominant 25
SLC26A4	605646	Deafness autosomal recessive 4 with enlarged vestibular aqueduct / Pendred syndrome
SMPX	300226	Deafness X-linked 4
STRC	606440	Deafness autosomal recessive 16
SYNE4	615535	Deafness autosomal recessive 76
TBC1D24	613577	Deafness autosomal recessive 86 / Deafness autosomal dominant 65
TECTA	602574	Deafness autosomal recessive 21 / Deafness autosomal dominant 8/12
TMC1	606706	Deafness autosomal recessive 7 / Deafness autosomal dominant 36
TMPRSS3	605511	Deafness autosomal recessive 8/10
TNC	187380	Deafness autosomal dominant 56
TPRN	613354	Deafness autosomal recessive 79
TRIOBP	609761	Deafness autosomal recessive 28
USH1C	605242	Deafness autosomal recessive 18A
USH1G	607696	Usher syndrome type 1G
USH2A	608400	Usher syndrome type 2A
WFS1	606201	Deafness autosomal dominant 6/14/38 / Wolfram syndrome 1
WHRN	607928	Deafness autosomal recessive 31 / Usher syndrome type 2D

Table S2. Pathogenic and likely pathogenic *ACTG1* variants reported in patients with Baraitser-Winter syndrome and/or associated congenital anomalies.

Exon [1]	Nucleotide change [1]	Protein change [1]	Protein subdomain [2]	Phenotype	Reference
2	c.34A>G	p.Asn12Asp	1	Baraitser-Winter syndrome	Di Donato et al. (2016) [47]
2	c.118C>T	p.His40Tyr	2	Baraitser-Winter syndrome	Posey et al. (2017) [54]; ClinVar database (Variation ID: 374385)
3	c.173C>T	p.Ala58Val	2	Baraitser-Winter syndrome; DFNA20/26 (?)	Kemerley et al. (2017) [52]
3	c.176A>G	p.Gln59Arg	2	Baraitser-Winter syndrome	Chacon-Camacho et al. (2020) [63]
3	c.209C>T	p.Pro70Leu	1	Ocular coloboma	Rainger et al. (2017) [55]
3	c.221G>T	p.Gly74Val	1	Baraitser-Winter syndrome	Gieldon et al. (2018) [64]
3	c.359C>T	p.Thr120Ile	1	Baraitser-Winter syndrome	Rivière et al. (2012) [6]; Verloes et al. (2015) [7]; Di Donato et al. (2014) [48]
4	c.404C>T	p.Ala135Val	1	Baraitser-Winter syndrome	Rivière et al. (2012) [6]; Verloes et al. (2015) [7]
4	c.439C>T	p.Arg147Cys	3	Baraitser-Winter syndrome	ClinVar database (Variation ID: 1012294)
4	c.459G>A	p.Met153Ile	3	Microlissencephaly; Baraitser-Winter syndrome (?)	Poirier et al. (2015) [49]
4	c.464C>T	p.Ser155Phe	3	Baraitser-Winter syndrome	Rivière et al. (2012) [6]; Verloes et al. (2015) [7]; Allawh et al. (2017) [51]
4	c.499G>A	p.Gly167Lys	3	Congenital diaphragmatic hernia, multiple minor anomalies, autism; Baraitser-Winter syndrome (?)	Longoni et al. (2017) [53]
4	c.574A>T	p.Ile192Phe	4	Multiple congenital anomalies; Baraitser-Winter syndrome (?)	Retterer et al. (2016) [50]
4	c.608C>A	p.Thr203Lys	4	Baraitser-Winter syndrome	Rivière et al. (2012) [6]; Verloes et al. (2015) [7]
4	c.608C>T	p.Thr203Met	4	Baraitser-Winter syndrome	Vontell et al. (2019) [60]; Chacon-Camacho et al. (2020) [63]
4	c.611C>G	p.Ala204Gly	4	Baraitser-Winter syndrome	ClinVar database (Variation ID: 452404)

Exon [1]	Nucleotide change [1]	Protein change [1]	Protein subdomain [2]	Phenotype	Reference
4	c.616C>T	p.Arg206Trp	4	Polymicrogyria; Baraitser-Winter syndrome (?)	Stutterd et al. (2021) [65]
4	c.628C>G	p.Arg210Gly	4	Baraitser-Winter syndrome	Yamamoto et al. (2019) [61]
4	c.628C>T	p.Arg210Cys	4	Baraitser-Winter syndrome	Thiffault et al. (2019) [59]
4	c.640G>A	p.Glu214Lys	4	Baraitser-Winter syndrome	Homma et al. (2019) [58]
4	c.728C>T	p.Pro243Leu	4	Baraitser-Winter syndrome; microlissencephaly	Poirier et al. (2015) [49]; ClinVar database (Variation ID: 807363)
4	c.760C>T	p.Arg254Trp	4	Baraitser-Winter syndrome	Rivière et al. (2012) [6]; Verloes et al. (2015) [7]; Di Donato et al. (2016) [47]; Di Donato et al. (2018) [57]
4	c.766C>T	p.Arg256Trp	4	Baraitser-Winter syndrome	Rivière et al. (2012) [6]; Verloes et al. (2015) [7]; Di Donato et al. (2016) [47]; Di Donato et al. (2018) [57]
4	c.767G>A	p.Arg256Gln	4	Baraitser-Winter syndrome	Accogli et al. (2020) [62]
4	c.773C>T	p.Pro258Leu	4	Baraitser-Winter syndrome	Zazo Seco et al. (2017) [56]; Perea-Romero et al. (2021) [66]
6	c.1000G>C	p.Glu334Gln	3	Baraitser-Winter syndrome	Di Donato et al. (2016) [47]
6	c.1004G>A	p.Arg335His	3	Baraitser-Winter syndrome	Di Donato et al. (2016) [47]

[1] Exon numbering and nucleotide changes of *ACTG1* gene was reported according to the RefSeq transcript NM_001614; the predicted protein change based on DNA data was reported according to the RefSeq protein NP_001605.1.
[2] Subdomain 1 (residues 1-32, 70-144, and 338-372), subdomain 2 (residues 33-69), subdomain 3 (residues 145-180, and 270-337), subdomain 4 (residues 181-269). [34, 35]

Table S3. *ACTG1* variants of uncertain significance reported in patients with Baraitser-Winter syndrome and/or associated congenital anomalies (only variants with MAF=0 in gnomAD v2.1.1 are reported).

Exon [1]	Nucleotide change [1]	Protein change [1]	Protein subdomain [2]	Phenotype	Reference
2	c.88G>T	p.Val30Leu	1	Pachygyria and corpus callosum partial agenesis; Baraitser-Winter syndrome (?)	Accogli et al. (2020) [62]
3	c.223A>C	p.Ile75Leu	1	Microlissencephaly; Baraitser-Winter syndrome (?)	Poirier et al. (2015) [49]
4	c.430G>A	p.Ala144Pro	1	Baraitser-Winter syndrome	ClinVar database (Variation ID: 434079)
4	c.485C>T	p.Thr162Met	3	Baraitser-Winter syndrome	ClinVar database (Variation ID: 1029360)
4	c.535G>T	p.Asp179Tyr	3	Baraitser-Winter syndrome	Di Donato et al. (2016) [47]; Di Donato et al. (2018) [57]
4	c.598T>A	p.Phe200Ile	4	Baraitser-Winter syndrome	ClinVar database (Variation ID: 128266)

[1] Exon numbering and nucleotide changes of *ACTG1* gene was reported according to the RefSeq transcript NM_001614; the predicted protein change based on DNA data was reported according to the RefSeq protein NP_001605.1.
[2] Subdomain 1 (residues 1-32, 70-144, and 338-372), subdomain 2 (residues 33-69), subdomain 3 (residues 145-180, and 270-337), subdomain 4 (residues 181-269). [34, 35]
MAF: minor allele frequency
gnomAD (genome aggregation database): https://gnomad.broadinstitute.org
ClinVar: https://www.ncbi.nlm.nih.gov/clinvar/
